# Antipruritic Effect of 2,3-Dehydrosilybin in Glial Cells and Chloroquine-Treated Mice

**DOI:** 10.4014/jmb.2502.02015

**Published:** 2025-05-28

**Authors:** Bo-Mi Kim, Byoung Ok Cho, Seon Il Jang

**Affiliations:** 1 Department of Chemical Engineering, Wonkwang University, Iksan 54538, Republic of Korea; 2 Institute of Health Science, Jeonju University, Jeonju 55069, Republic of Korea; 3 Department of Health Management, Jeonju University, Jeonju 55069, Republic of Korea

**Keywords:** Pruritus, 2,3-dehydrosilybin, IL-31, STAT3, LCN2

## Abstract

Pruritus is an inflammatory skin disorder that reduces the patient’s quality of life. Meanwhile, 2,3-dehydrosilybin (DHS) belongs to a class of flavonolignans derived from milk thistle seeds, and is known to have anticancer, hepatoprotective, antioxidant, and angiogenic effects. In the current study, the inhibitory effect of DHS on pruritus was investigated in lipopolysaccharide (LPS)-stimulated microglia, IL-31- and IL-6-treated astrocytes, and chloroquine-treated mice. DHS was shown to suppress pruritus-related cytokines IL-31 and IL-6 in LPS-treated microglia. Moreover, DHS inhibited activation of MAPKs (p38, ERK, and JNK) in LPS-stimulated microglia. Furthermore, DHS prevented activation of STAT3 and LCN-2 production in IL-31- and IL-6-treated astrocytes. In addition, DHS inhibited scratching and GFAP expression in chloroquine-treated mice. These results suggest that DHS may prevent and/or treat pruritus.

## Introduction

Pruritus is a common symptom of various inflammatory skin conditions in which the underlying mechanism is not well addressed [1]. Interleukin (IL)-31 is an inflammatory cytokine that is structurally similar to the IL-6 family cytokines. It is thought to be an important factor among the mediators that induce pruritus in atopic dermatitis (AD) [1]. In addition to the key role of IL-31 in pruritus, large amounts of IL-31 were also found in the serum and tissues of patients with inflammatory bowel disease, Crohn’s disease, rhinitis, and asthma [2, 3]. The existence of IL-31 receptors (IL-31RA and OSMR β) in the brain tissues and spinal cord, and their association with itch, raises the possibility that cells of the central nervous system (CNS) can mediate itch by directly stimulating sensory neurons through inducing IL-31 [4]. Moreover, it was recently determined that astrocytes in the dorsal horn of the spine are responsive in atopic and contact dermatitis models through the activation of signal transducer and activator of transcription 3 (STAT3), and contribute crucially to itch [5]. Astrocytes and microglia form the most abundant cell populations in the CNS [6]. These cells can secrete various biologically active substances that can stimulate other cells [6, 7]. In inflammatory conditions, astrocytes and microglia are activated and release pro-inflammatory cytokines, such as tumor necrosis factor (TNF)-α and IL-1β [8]. In neurological disorders like multiple sclerosis, parkinson’s disease (PD), HIV-1-associated dementia (HAD), and alzheimer’s disease, activated astrocytes secrete pro-inflammatory mediators (various cytokines and chemokines) by activating NF-κB and STAT3 pathways, causing cell injury in the brain [9, 10]. Recently, astrocytes and microglia have come to be recognized as novel players in pruritus in AD; however, it is not yet known what biologically active substances they secrete to promote pruritus in AD [11].

**Fig. 1:**
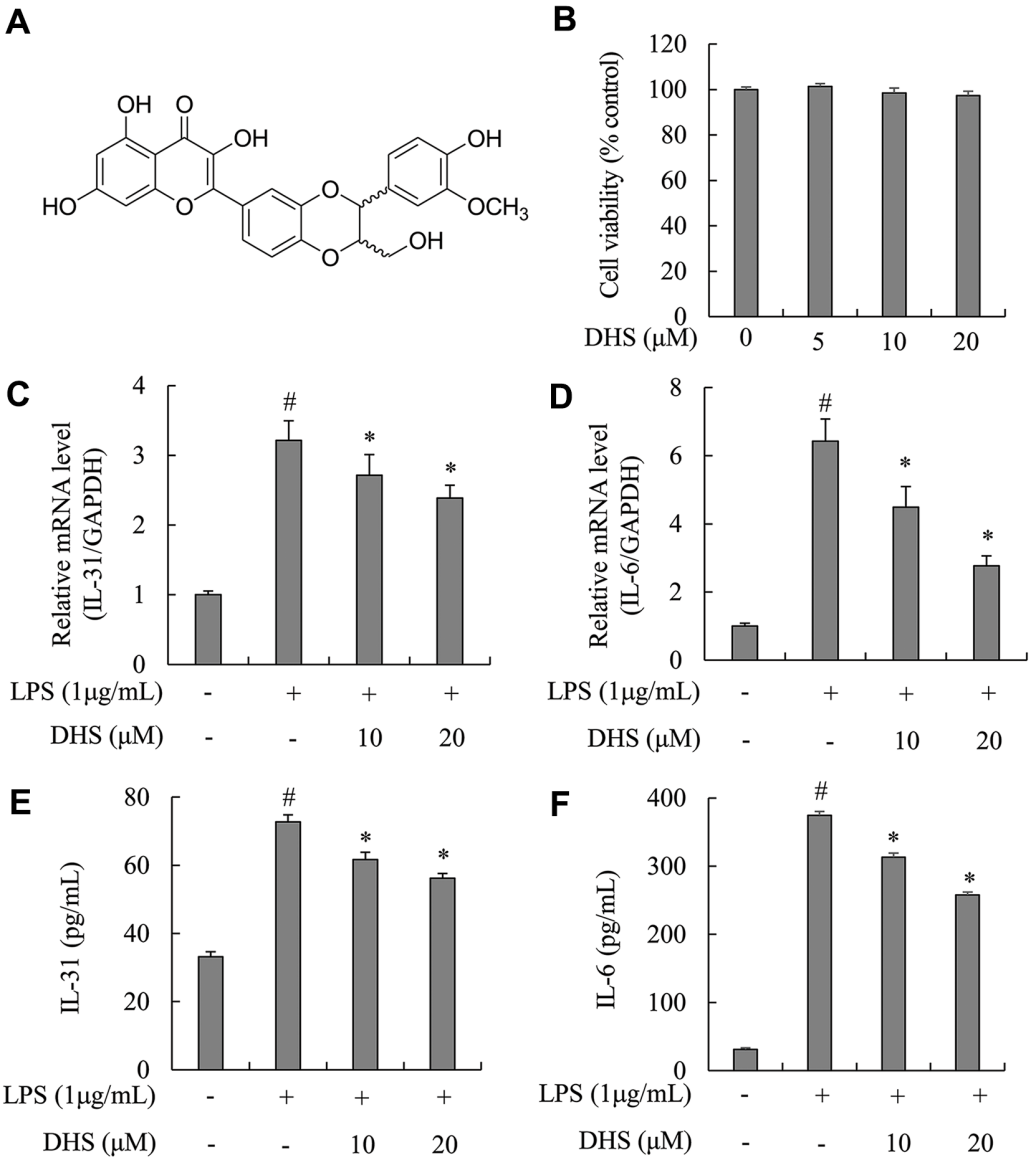
Effects of DHS on IL-31 and IL-6 expression in LPS-treated microglia. (**A**) Structure of DHS. (**B**) Cell toxicity of DHS in microglia. (**C** and **D**) Real-time PCR result. (**E** and **F**) ELISA result. Microglia were pre-treated with DHS (10 and 20 μM) for 1 h and subsequently stimulated with 1 μg/ml of LPS for 3 h or 24 h. The expression levels of IL-31 and IL-6 mRNA were determined by RT-PCR. The levels of IL-31 and IL-6 secretion were measured by ELISA assay. Each bar represents the mean ± SD. ^#^*p* < 0.05 vs. untreated cells. *p < 0.05 vs. LPS-stimulated cells.

The flavonolignan 2,3-dehydrosilybin (DHS, Fig. 1A), derived from milk thistle seeds, has been reported to have anticancer, hepatoprotective, angiogenic, and antioxidant effects [12-14]. However, the effect of DHS on the activation mechanism of IL-31 and STAT3 in cells and itch model mice is not well understood. To determine the effect and mechanism of DHS on pruritus, we conducted preliminary research on the signal pathways of DHS in microglia and astrocytes. On this basis, we examined the effect of DHS on pruritus in chloroquine-injected mice.

## Materials and Methods

Murine astrocytes (C8-D1A, CRL-2541) and microglial cells (SIM-A9, CRL-3265) were purchased from ATCC (USA). Fetal bovine serum (FBS), Iscove’s Modified Dulbecco’s Medium (IMDM), Dulbecco’s modified Eagle medium (DMEM), horse serum (HS), radio-immunoprecipitation assay buffer (RIPA buffer), penicillin/streptomycin, Protease and Phosphatase Inhibitor Cocktail, IL-31 an enzyme-linked immunosorbent assay (ELISA) kit, goat anti-mouse IgG Alexa Fluor 488, and glial fibrillary acidic protein (GFAP) antibody were acquired from Thermo Fisher Scientific (USA). DHS was obtained by γ-irradiation as previously described [15]. EZ-Cytox WST-based Cell Viability and EZ-Western Lumi Femto kits were procured from DAEIL LAB Service (Republic of Korea). An RNA-Spin Total RNA Extraction Kit was procured from iNtRON Biotechnology (Republic of Korea ). SYBR Premix Ex Taq was obtained from Toyobo (Japan). Bradford’s assay reagent, an iScript cDNA Synthesis Kit, premixed protein sample buffer, and polyvinylidene fluoride (PVDF) membrane were purchased from Bio-Rad Laboratories (Hercules, USA). An ELISA kit for IL-6, recombinant IL-6 protein, and recombinant IL-31 protein were obtained from R&D Systems (USA). Lipopolysaccharides (LPS), dimethyl sulfoxide (DMSO), and chloroquine diphosphate salt (CQ) were obtained from Sigma-Aldrich (USA). Antibodies for phospho (p)-ERK, ERK, p-JNK, JNK, p-p38, and p38 mitogen-activated protein kinases (MAPKs) were obtained from Santa Cruz Biotechnology (USA). STAT3, p-STAT3, β-actin, and horseradish peroxidase (HRP)-IgG secondary antibodies were obtained from Cell Signaling Technology (USA). A hematoxylin and eosin (H&E) staining kit was obtained from Abcam (UK).

### Cell Culture and Cell Viability

Microglial cells were cultured in IMDM supplemented with 10% FBS, 5% HS, and 1% penicillin/streptomycin in an incubator under conditions of 37°C and 5% CO_2_. Astrocytes were cultured in DMEM supplemented with 10% FBS, 5% HS, and 1% penicillin/streptomycin in an incubator at 37°C and 5% CO_2_. The microglia and astrocytes were cultured to 80% confluency before subsequent experiments. The cells were not serum starved in any of the experiments.

For cell viability studies, the microglia (3 × 10^5^ cells/ml) and astrocytes (1 × 10^5^ cells/ml) were cultured in a 96-well culture plate for 16 h and incubated in 0, 5, 10, and 20 μM of DHS for 72 h. After 72 h, 0.01 mL of EZ-Cytox reagent was added to each well and incubated for 4 h. The cell viability was identified by measuring the absorbance of each well at 450 nm with a microplate reader (Tecan, Switzerland). The absorbance was correlated with the number of live cells.

### Cell Treatment

After being cultured to 80% confluency, microglia (3 × 10^5^ cells/ml) were pre-treated with 10 μM and 20 μM of DHS for 1 h, before being treated with 1 μg/ml of LPS. After LPS stimulation, the cells were cultured for either 0.5 h, 3 h, or 24 h depending on the experiment to be conducted. Astrocytes (1 × 10^5^ cells/ml) were cultured to 80%confluency and pre-treated with 10 μM and 20 μM of DHS for 1 h before stimulation with 10 ng/ml of IL-6 or IL-31. After stimulation with IL-6 or IL-31, the cells were cultured for either 3 h or 0.5 h depending on the experiment to be conducted. Cells without DHS pre-treatment and stimulation, and cells without DHS pre-treatment but stimulated with LPS, were incubated in parallel as negative or positive controls.

### Real-Time (RT) Quantitative PCR Analysis

Microglia pre-treated with or without DHS for 1 h were treated with LPS for 3 h, and astrocytes pre-treated with or without DHS for 1 h were treated with IL-6 or IL-31 for 4 h. Total RNA from cultured microglia and astrocytes was extracted and purified using a commercial RNA extraction kit (iNtRON Biotechnology) according to the manufacturer’s protocols. The total RNA concentration was confirmed by spectrophotometry. Each sample (1 μg) was reverse transcribed to cDNA using an iScript cDNA Synthesis Kit (Bio-Rad) and a T100 Thermal Cycler (Bio-Rad). Real-time PCR amplification for lipocalin-2 (LCN2), IL-6, IL-31, and GAPDH was conducted using SYBR Premix Ex Taq (Toyobo) and a StepOne Real-Time PCR System (Thermo Fisher Scientific) under the following conditions: 95°C for 3 min and amplification (40 cycles) at 95°C for 30 s, 60°C for 30 s, and 72°C for 30 s. The primer pairs used for IL-6 were 5’-TCC AGT TGC CTT CTT GGG AC-3’ (forward) and 5’-ACA GGT CTG TTG GGA GTG GT-3’ (reverse); those for IL-31 were 5’-CCT ACC CTG GTG CGT CTT TG-3’ (forward) and 5’-CTG ACA TCC CAG ATG CCT GC-3’ (reverse); those for LCN2 were 5'-CCA GTT CGC CAT GGT ATT TT-3' (forward) and 5'-GGT GGG GAC AGA GAA GAT GA-3' (reverse); and those for GAPDH were 5’-GGC TAC ACT GAG GAC CAG GT-3’ (forward) and 5’-TCC ACC ACC CTG TTG CTG TA-3’ (reverse). Expression levels were normalized to GAPDH using the 2^-ΔΔCt^ comparative method.

### ELISA Assay for IL-6 and IL-31

For the ELISA, 50 μl of culture supernatants from microglia treated with or without DHS and treated with LPS for 24 h were used. The levels of IL-6 and IL-31 cytokine secreted from microglia were determined with an ELISA kit provided by a commercial company and used in accordance with the manufacturer’s directions.

### Protein Extraction

The cell protein extraction was performed according to the manufacturer’s directions using RIPA buffer. In brief, microglia and astrocytes were rinsed in cold phosphate-buffered saline (PBS) and collected from the culture dish. The cells were then pelleted by centrifugation at 1,500 ×*g* for 5 min in 15 ml conical tubes. Following that, the pellet was washed twice with cold PBS. RIPA buffer (100 μl) with protease and phosphatase inhibitor cocktails was then mixed with the pelleted cells to suspend the cell pellet, which was kept on ice for 20 min and vortexed at 5 min interval. The mixture was then centrifuged at 14,000 ×*g* for 15 min. The protein sample from each tube was transferred to a fresh tube and stored at -80°C for further analysis.

### Western Blotting

After quantitative analysis of proteins utilizing the Bradford’s assay reagent, each sample was applied to 12% or 10% SDS-PAGE (90 min, 110 V). The seperated protein samples were transferred to the PVDF membrane (1 h, 100 V). Then, the membrane was cultivated in 5% non-fat dry milk (Bio-Rad) for 60 min and subsequently incubated with various primary antibodies (namely, p-ERK, ERK, p-JNK, JNK, p-p38, p38, p-STAT3, STAT3, and β-actin) at 4°C for 18 h. After the above incubation, the membrane was washed three times (10 min each) with Tris-buffered saline involving 0.01% Tween-20 (TBST), and then incubated with the corresponding HRP-conjugated secondary antibody for 120 min at room temperature. Subsequently, the membrane was washed six times utilizing TBST (5 min each time), and the chemiluminescence reagent of an EZ-Western Lumi Femto Kit together with an imaging system (Alliance version 15.11; UVITEC Cambridge, UK) were utilized to acquire the images. Band densities were determined using ImageJ version 1.52 (NIH, USA).

### Animals and Treatments

Pathogen-free, four-week-old ICR male mice (weight: 19 ± 1 g) from Orient Bio Inc. (Republic of Korea ) were placed in a room at 55 ± 5% humidity and 20-24°C, with a light and dark cycle of 12/12 h, and free access to food and water. The procedures for this animal experiment were conducted according to the instructions of the Jeonju University Institutional Animal Care and Used Committee (Approval No. jjIACUC-20230602-2022-0501-A1). Mice were randomly divided into the following three groups with 5 mice in each group: group 1, normal control; group 2, 50 μg/site of chloroquine (called positive control); and group 3, 50 μg/site of chloroquine plus 20 mg/kg of DHS (DHS could be created in vehicle) (0.05% DMSO and Tween-20). The mice were intraperitoneally administered with 20 mg/kg of body weight of DHS or vehicle before chloroquine injection. One hour later, to induce pruritus in groups 2 and 3, 50 μg/site (0.1 ml) of chloroquine was injected subcutaneously into the left shoulder of mice. After chloroquine injection, scratching behavior in mice was monitored with micro cameras according to Mihara *et al*.'s protocol [16], (ONCCTV, Republic of Korea) for 1 h, and the number of scratches on the site of the chloroquine injections was counted in a double-blinded manner. All groups of mice were subsequently euthanized. Back skin samples were then obtained for histopathological detection and vertebrae were obtained for immunofluorescence staining.

### Histopathological Examination

The back skin tissues were fixed with neutral buffered formalin, washed in cold PBS, and then dehydrated in a sequence of graded ethanol. Following that, the tissues were cleared in xylene and embedded in paraffin, and a rotary microtome (Leica, Germany) was then utilized to cut the tissues into sections (5 μm thick). H&E staining was applied to the sections to assess histopathological changes. All of the staining schemes were performed with little or no modification. An optical microscope (Leica) was utilized to examine histopathological changes.

### Immunofluorescence for GFAP

The fifth lumbar segments were fixed in a mixture of 4% paraformaldehyde at 37°C for 5 h and placed in PBS containing 30% sucrose for 24 h at 4°C. Samples were cut into 30 μm thick sections by a cryotome (Amos Scientific Pty, Ltd., Australia). The samples were then washed six times for 5 min each with PBS and placed in PBS containing 0.3% Triton X-100 (Sigma-Aldrich) and 2% bovine serum albumin (Sigma-Aldrich) for 1 h at room temperature. Samples were incubated with GFAP antibody at 4°C for 18 h. After washing three times with PBS for 10 min each, samples were incubated with the goat anti-mouse IgG Alexa Fluor 488 secondary antibody for 2 h at room temperature. Samples were then washed with PBS again and mounted on glass slides. After drying for 20 h, innunofluorescence images were captured at magnification of ×400 using a fluorescence microscope (Carl Zeiss AG, Germany).

### Statistical Analysis

The statistical analyses were performed using the SPSS statistics program (version 22 SPSS Inc., USA). One-way analysis of variance (ANOVA) was applied to report the differences between variables. The average values were comparaed via Tukey's post hoc test (*p*-value < 0.05). All data are described with the mean ± SD.

## Results

### DHS Blocked IL-31 and IL-6 mRNA Expression and Secretion in LPS-Treated Microglia

First, the cytotoxic activity of DHS at various concentrations was estimated after treatment for 72 h. With the concentration between 0 and 20 μM, the cell viability assay showed no cytotoxicity (Fig. 1B). Based on these findings, DHS concentrations of 10 and 20 μM were employed in later experiments. To explore effect of DHS on IL-31 and IL-6 mRNA expression and secretion related to pruritus in LPS-treated microglia, PCR and ELISA assays were conducted. As illustrated in Fig. 1C, 1D, 1E, and 1F, mRNA expression and secretion of IL-31 and IL-6 cytokines were notably increased in LPS-treated microglia. However, pre-treatment with DHS resulted in significant decreases of mRNA expression and secretion of IL-31 and IL-6 cytokines.

### DHS Inhibited Activation of MAPKs in LPS-Treated Microglia

**Fig. 2:**
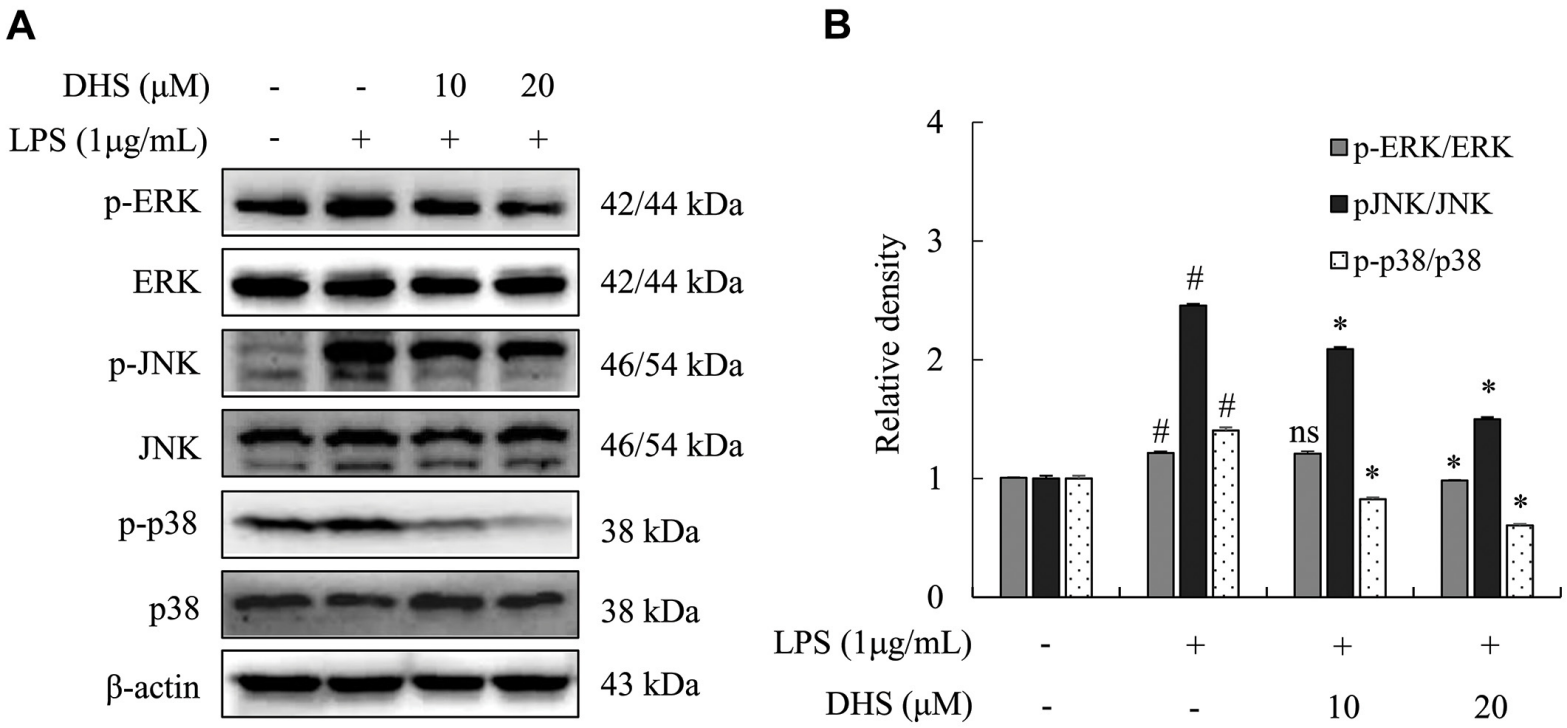
Effects of DHS on activation of MAPKs in LPS-treated microglia. Microglia were pre-treated with DHS (10 and 20 μM) for 1 h and subsequently stimulated with 1 μg/ml of LPS for 30 min. (**A**) The protein expression levels of cell signaling kinases were investigated via western blotting assay. (**B**) ImageJ analysis software was used to analyze the densities of each band with non-phosphorylated counterparts. Each bar represents the mean ± SD. ^#^*p* < 0.05 vs. untreated cells. *p < 0.05 vs. LPS-stimulated cells. ns, no significant difference compared to LPS-treated cells.

To explain the mechanism by which DHS inhibits IL-31 and IL-6 production, we investigated the phosphorylation of MAPKs (ERK, JNK, and p38) in LPS-treated microglia. The MAPK pathway plays a key role in the control of inflammatory responses, including the production of cytokines like IL-31 and IL-6. Phosphorylation of these MAPKs is often a principal step in the activation of inflammatory cascades [17, 25]. In this study, LPS stimulation for 30 min led to the phosphorylation of ERK, JNK, and p38. Pre-treatment with DHS significantly inhibited the phosphorylation of these MAPKs in stimulated microglia (Fig. 2).

### DHS Suppressed STAT3 Activation and LCN2 mRNA Levels in Stimulated Astrocytes

**Fig. 3:**
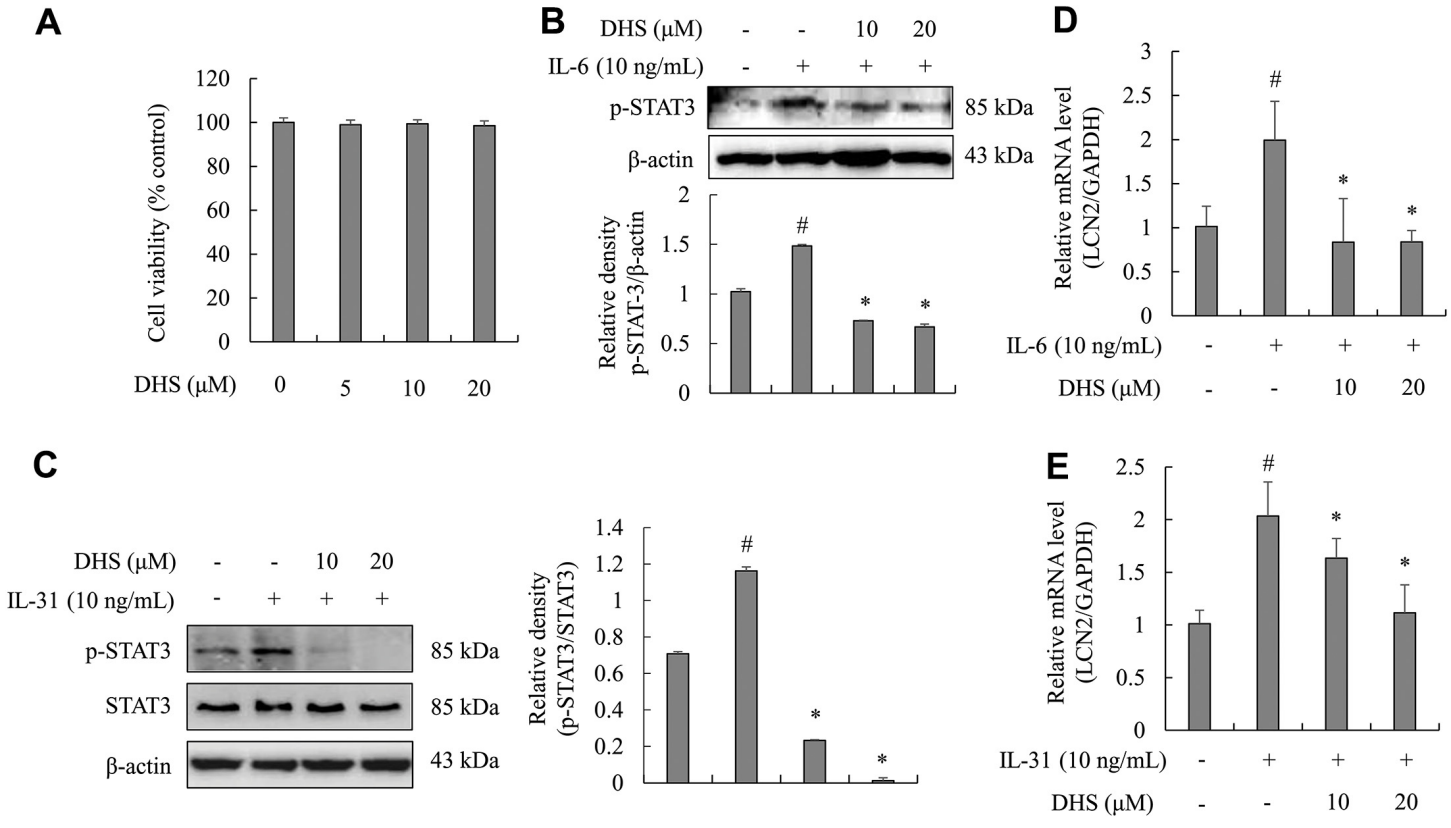
Effects of DHS on activation of STAT3 and LCN2 mRNA expression in IL-6 and IL-31-treated astrocytes. (**A**) Cell toxicity of DHS in astrocytes. (**B-E**) Astrocytes were pre-treated with DHS (10 and 20 μM) for 1 h and subsequently stimulated with 10 ng/ml of IL-6 and IL-31 for 30 min (**B** and **C**) or 4 h (**D** and **E**). The phosphorylation levels of STAT3 were investigated via western blotting assay. ImageJ analysis software was used to analyze the densities of each band. The expression levels of LCN2 mRNA were determined by RT-PCR. Each bar represents the mean ± SD. ^#^*p* < 0.05 vs. untreated cells. *p < 0.05 vs. IL-6-stimulated cells.

In astrocytes, the cytotoxic activity of DHS at various concentrations was tested. The results showed DHS as having no cytotoxicity (Fig. 3A). To prove the effect of DHS on STAT3 activation and LCN2 expression, western blotting and PCR were performed in IL-6- and IL-31-stimulated astrocytes, respectively. As illustrated in Fig. 3B and 3C, IL-6 and IL-31 stimulation significantly promoted the activation of STAT3 in astrocytes. However, pre-treatment of DHS inhibited IL-6- and IL-31-induced activation of STAT3. In addition, IL-6 and IL-31 stimulation significantly promoted the LCN2 mRNA expression levels in astrocytes. However, pre-treatment of DHS inhibited the IL-6- and IL-31-induced mRNA levels of LCN2 (Fig. 3D and 3E).

### DHS Inhibited Chloroquine-Induced Scratching Behavior and Histopathological Changes in Mice

**Fig. 4:**
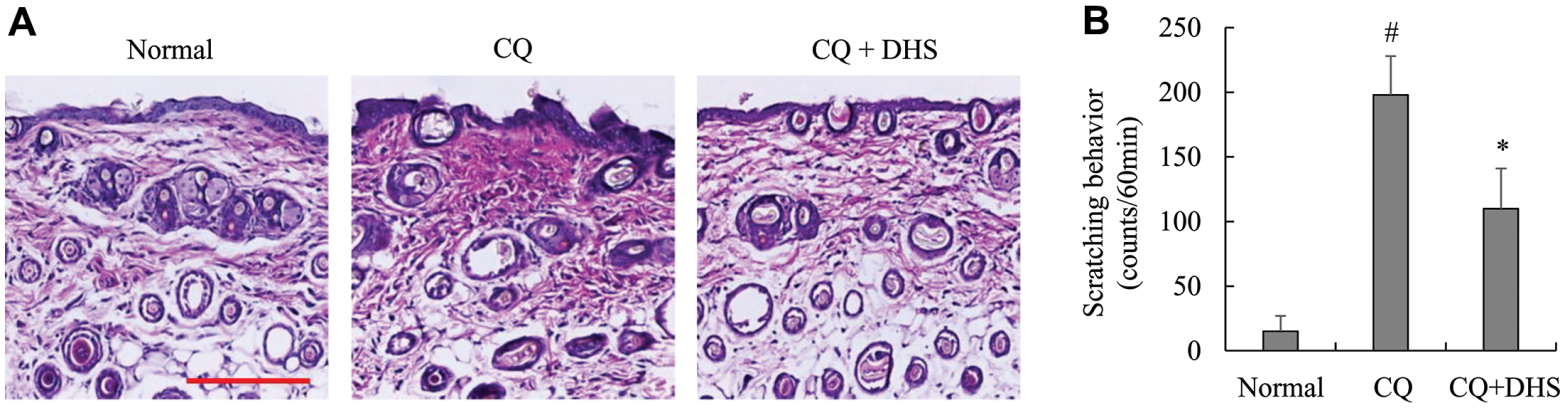
Effects of DHS on chloroquine-induced scratching behavior and histological changes. (**A**) The skin sections were stained with hematoxylin and eosin. Sections were evaluated using microscope at a magnification of ×100 (scale bar, 200 μm). (**B**) Scratching behavior was measured by a double-blind test. Each bar represents the mean ± SD. ^#^*p* < 0.05 vs. untreated cells. *p < 0.05 vs. CQ-injected group. CQ, chloroquine.

The anti-pruritic effect of DHS was determined in a chloroquine-induced pruritus model in ICR mice. As shown in Fig. 4A, histopathological changes such as epidermal thickness and edema were observed in skin tissues from the site of chloroquine injections. However, when mice were pre-treated with DHS before chloroquine injections, recovery in histological changes was observed. Additionally, the control mice pre-treated with saline showed limited scratching behavior, whereas ICR mice injected subcutaneously with chloroquine showed intense scratching behavior (Fig. 4B). Intraperitoneal pre-treatment with DHS (20 mg/kg) significantly suppressed chloroquine-induced scratching (Fig. 4B).

### Effect of DHS on GFAP Activity in Chloroquine-Injected Mice

**Fig. 5:**
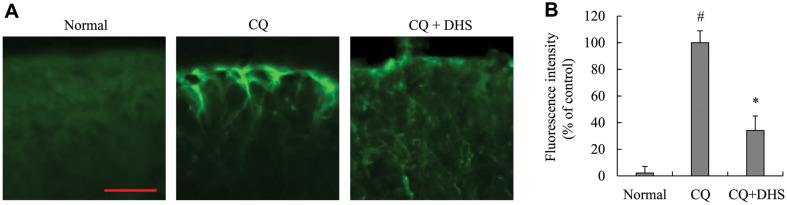
Effects of DHS on SDH astrocyte activity in chloroquine-injected mice. The SDH astrocyte activity was evaluated by GFAP-specific innunofluorescence assay. The stained spinal tissues were observed with microscope at a magnification of ×400 (scale bar, 40 μm). Each bar represents the mean ± SD. ^#^*p* < 0.05 vs. untreated cells. *p < 0.05 vs. CQinjected group. CQ, chloroquine.

To elucidate the effect of DHS on mouse spinal dorsal horn (SDH) astrocyte activity, a GFAP-specific innunofluorescence assay was carried out in mice spinal sections. As shown in Fig. 5, the expression of GFAP in spinal sections of chloroquine-injected mice was meaningfully increased, whereas DHS administration significantly prevented GFAP expression induced by chloroquine.

## Discussion

In previous studies, we reported that LPS induces IL-31 and IL-6 cytokines in activated astrocytes and microglia [17, 18]. In addition, IL-31 is directly involved in pruritus found in AD patients as it is exhibited more in AD mice undergoing pruritus than in AD mice without pruritus [19]. It has been reported that IL-6 upregulation is associated with pruritus in the skin of uremic pruritus patients [20]. DHS is known to have anticancer, anti-hepatoprotective, anti-angiogenic and antioxidant activities by inhibiting inflammatory processes related to these diseases [12-14]. However, research on the efficacy of DHS in controlling IL-31, IL-6, and pruritus is insufficient. Therefore, the current study aimed to evaluate the efficacy of DHS on LPS-induced microglia activation and IL-6-and IL-31-induced astrocyte activation, and also to determine the efficacy of DHS on chloroquine-induced pruritus in mice.

First, we determined whether the activated microglia produce IL-31 and IL-6, which are important cytokines involved in the pathogenesis of some disorders in CNS. Microglia interact with neurons in chronic itch to promote its persistence [21]. Therefore, modulating microglial inflammation may present a promising target for managing chronic itch. Although little research has been done on IL-31 production in CNS, IL-31 receptors (IL-31RA and OSMR β) have been extensively found in the brain tissues and spinal cord and IL-31 is acknowledged as causing pruritus in AD [22, 23]. IL-6 is secreted by immune cells and acts as a link for contact between the nervous and immune system and causes itching, although direct evidence is currently limited [24]. Therefore, suppressing the production of cytokine in CNS may be beneficial in the treatment or prevention of various CNS-related disorders. We examined the expression and secretion of IL-31 and IL-6 in microglia and our findings suggest that the elevation of IL-31 and IL-6 levels in CNS could play a direct role in exciting other cells of CNS to cause pruritus.

The MAPK signaling cascade is a pathway of the immune response that plays a crucial role in intracellular signal responses and modulates the expression of a number of cytokines [25]. Many studies have demonstrated that the neuroinflammatory response is related to the regulation of the MAPK cascade in LPS-stimulated microglia [17, 26]. This makes MAPK a key target for treatment of allergic and inflammatory disorders. Thus, to better comprehend the mechanism of action underlying the inhibitory effects of DHS on IL-31 and IL-6 secretion, the regulatory effect of DHS on the activation of MAPKs in microglia was determined, and the results showed that DHS modulated the inhibition of ERK, JNK, and p38 MAPK activation in microglia. This result is in agreement with previous experiment [17].

Some activated astrocytes may regenerate tissue, foster neuronal survival, and rebuild damaged blood-brain barriers, but some activated astrocytes can release signaling molecules, causing side effects such as neuronal cell death and chronic pain [27]. Reactive astrocytes in spinal cord have also been proposed as novel players in chronic pruritus, but it is still largely unknown what biologically active substances they secrete to promote pruritus. Meanwhile, STAT3 and TLR4 receptors have been demonstrated to be extensively induced in reactive astrocytes [11]. Therefore, inhibition of astrocyte activation is necessary to treat several brain disorders as well as itch, PD, spinal cord injury, and other glia-mediated neuro-inflammatory diseases [11, 28, 29].

Itch is an inflammatory skin disease, and recent research has demonstrated that astrocytes in dorsal horn of spine are triggered in contact dermatitis and AD models by regulating STAT3 activation, and contribute critically to itch [5]. Reactive astrocytes enhance itch by stimulating the signaling system of the gastrin-releasing peptide (GRP) and its receptor (GRPR) through secretion of LCN2 in skin disease models [30]. Persistent activation of astrocytic STAT3 contributes to the elevation of pruritus. STAT3 activation increases LCN2 expression and enhances pruritus in astrocytes [31]. We therefore investigated the suppression of LCN2 expression and STAT3 activation by DHS in astrocytes. Our data showed that DHS prevented STAT3 activation and decreased the levels of LCN2 mRNA expression in IL-6- and IL-31-induced astrocytes. Therefore, DHS could reduce itching by preventing the levels of LCN2 mRNA via the STAT3 signaling pathway. Our recent study demonstrated that microglia-secreted IL-6 and IL-31 cytokines induce astrocyte activation via STAT3 activation and LCN2 expression [32], suggesting that the present results are consistent with our past experiments. Our findings indicate that the inhibitory effects of DHS could attenuate CNS-related pruritus by regulating the activity of microglia and astrocytes.

**Fig. 6:**
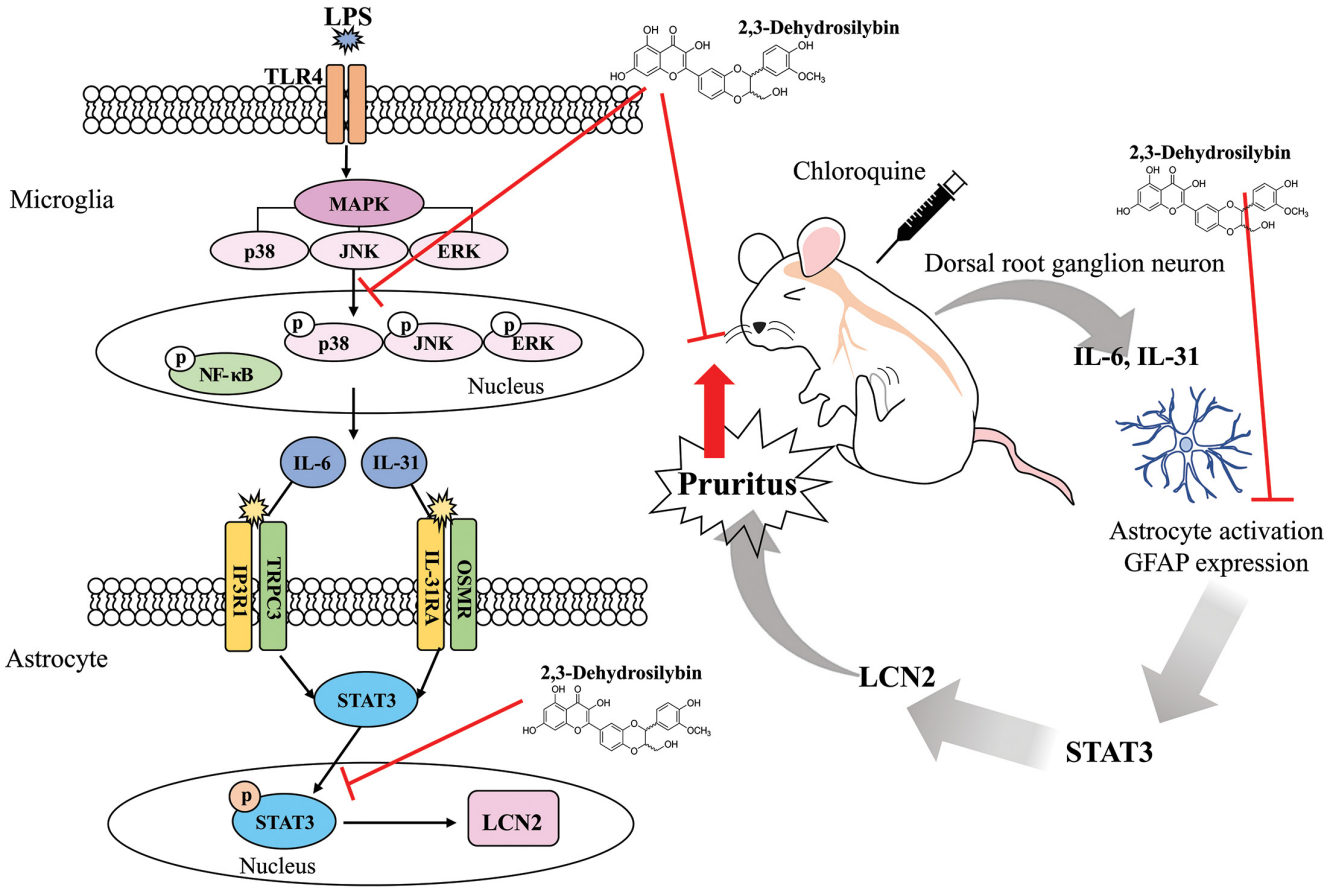
Schematic diagram of the proposed mechanism involved in the anti-pruritic effects of DHS.

Further research will be needed to confirm these facts in animal models. Chloroquine is known to cause itching via the GRP/GRPR signaling pathway in a histamine-independent manner [33]. Therefore, we investigated whether DHS suppressed chloroquine-induced itch in mice in vivo. Our data showed that DHS prevented chloroquine-induced scratching behavior and reduced histopathological changes in chloroquine-injected mice skin. Several studies have reported that pruritus-induced mice show increased GFAP expression in the SDH [5, 30, 33, 34]. Next, we investigated GFAP, a marker of astrocyte activation. We found that DHS inhibited GFAP expression, thus indicating that DHS inhibits astrocyte activation in chloroquine-treated mice, thereby reducing pruritus. The anti-pruritic effect of DHS can be seen as reducing the glial cell activation through the reduction of cytokines and inflammatory reactions. We assume that the inhibitory activity of DHS might be due to the regulation of astrocyte activity by inhibiting STAT3 activation, as depicted in Fig. 6. However, the current study did not show that the suppressive effect of DHS on astrocyte activation in vivo is a result of STAT3 and LCN2 inhibition as well as microglia relevance. Therefore, future research will be needed to clarify the precise inhibitory mechanisms of DHS in pruritus models.

## Conclusion

DHS suppressed the elevation of IL-6 and IL-31 cytokines through the suppression of MAPK activation in LPS-activated microglia, and suppressed STAT3 activation and LCN2 mRNA expression in IL-6- and IL-31-stimulated astrocytes. DHS also reduced scratching behavior and histopathological changes in chloroquine-injected mice skin. In addition, DHS inhibited the expression of GFAP in chloroquine-injected mice spinal cord. Thus, the current study provides new evidence that DHS may significantly contribute to the prevention or treatment of microglia and astrocyte-induced pruritus that is not ameliorated by histamine blockers alone.
